# Identification of Molecular Signatures and Candidate Drugs in Vascular Dementia by Bioinformatics Analyses

**DOI:** 10.3389/fnmol.2022.751044

**Published:** 2022-02-11

**Authors:** Jun Shu, Wenshi Wei, Li Zhang

**Affiliations:** Department of Neurology, Cognitive Disorders Center, Huadong Hospital, Fudan University, Shanghai, China

**Keywords:** systems biology, gene–drug interaction, hub gene, vascular dementia, protein–protein interaction (PPI)

## Abstract

Vascular dementia (VaD) is considered to be the second most common form of dementia after Alzheimer’s disease, and no specific drugs have been approved for VaD treatment. We aimed to identify shared transcriptomic signatures between the frontal cortex and temporal cortex in VaD by bioinformatics analyses. Gene ontology and pathway enrichment analyses, protein–protein interaction (PPI) and hub gene identification, hub gene–transcription factor interaction, hub gene–microRNA interaction, and hub gene–drug interaction analyses were performed. We identified 159 overlapping differentially expressed genes (DEGs) between the frontal cortex and temporal cortex that were enriched mainly in inflammation and innate immunity, synapse pruning, regeneration, positive regulation of angiogenesis, response to nutrient levels, and positive regulation of the digestive system process. We identified 10 hub genes in the PPI network (*GNG13*, *CD163*, *C1QA*, *TLR2*, *SST*, *C1QB*, *ITGB2*, *CCR5*, *CRH*, and *TAC1*), four central regulatory transcription factors (FOXC1, CREB1, GATA2, and HINFP), and four microRNAs (miR-27a-3p, miR-146a-5p, miR-335-5p, and miR-129-2-3p). Hub gene–drug interaction analysis found four drugs (maraviroc, cenicriviroc, PF-04634817, and efalizumab) that could be potential drugs for VaD treatment. Together, our results may contribute to understanding the underlying mechanisms in VaD and provide potential targets and drugs for therapeutic intervention.

## Introduction

Vascular dementia (VaD) is a neurocognitive disorder that also has other characteristics such as behavioral symptoms, locomotor abnormalities, and autonomic dysfunction (O’Brien and Thomas, 2015). Various clinical and population studies have indicated that the prevalence of early-onset VaD (<65 years old) ranges from 3.1 to 44% (Vieira et al., 2013). VaD is considered to be the second most common form of dementia after Alzheimer’s disease (AD), posing a heavy burden on families and societies (Khan et al., 2016). However, no specific drugs have been approved for VaD treatment. Growing evidence indicates that stroke, hypertension, diabetes, and atherosclerosis are risk factors for VaD (Kalaria, 2018). A recent study found that the incidence of post-event dementia at 1 year was 34⋅4% in patients with severe stroke, 8⋅2% in patients with minor stroke, and 5⋅2% in patients with transient ischemic attack (Pendlebury and Rothwell, 2019). The underlying mechanism of VaD is still largely unknown but may be associated with pathological processes including chronic hypoperfusion and hypoxia (Tukacs et al., 2020), vascular endothelial dysfunction and damage (Wang et al., 2018), increased blood–brain barrier permeability (Ueno et al., 2019), and oxidative stress and inflammation (Guo et al., 2020; Wang et al., 2020). Therefore, there is a need to elucidate the detailed molecular mechanisms of VaD and explore potential drugs for VaD treatment.

Bioinformatics analysis of transcriptomic data is used widely to identify biomarkers, therapeutic targets, and the mechanisms of various diseases, including dementia (Ma et al., 2019). Key modules and hub genes related to AD have been identified (Rahman M. R. et al., 2020; Lee and Lee, 2021), yet VaD has received little attention. A vascular basis for neuronal atrophy in both the temporal and frontal lobes in VaD that is entirely independent of any Alzheimer’s pathology has been reported (Kalaria, 2016). Therefore, in this study, we analyzed transcriptome signatures of the temporal cortex and frontal cortex samples from a microarray dataset of VaD to identify overlapping differentially expressed genes (DEGs) between the two brain regions. Gene ontology and pathway enrichment analyses were conducted to predict the biological activities of the overlapping DEGs. Protein–protein interaction (PPI) analysis was performed to detect potential relationships among the proteins encoded by the overlapping DEGs. Hub genes and key modules were identified using Cytoscape software. We also performed hub gene–transcription factor (TF) and hub gene–microRNA (miRNA) interaction analyses to detect potential transcriptional and posttranscriptional regulatory factors, and hub gene–drug interaction analysis to identify candidate drugs. These inclusive systems biology processes allowed us to identify potential biomolecular signatures of VaD, which will give new insights into the underlying mechanisms and provide potential targets for therapeutic intervention.

## Materials and Methods

**FIGURE 1:**
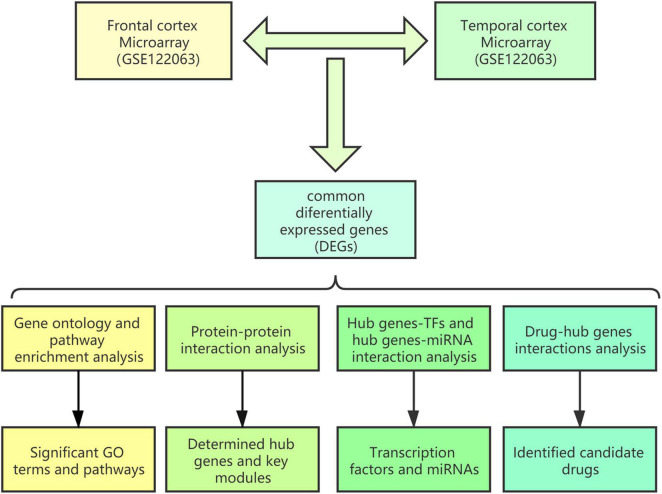
Schematic diagram of the workflow of the bioinformatics analyses conducted in this study.

A schematic diagram of the workflow of the bioinformatics analyses is shown in Figure 1.

### Identification of DEGs From Microarray Datasets of the Frontal Cortex and Temporal Cortex Samples From Patients With VaD

We used the Gene Expression Omnibus (GEO) dataset, GSE122063, which contains gene expression profiles of the frontal cortex and temporal cortex samples from patients with AD, VaD, and matched controls (McKay et al., 2019). In the current study, we analyzed only the gene expression data of the frontal cortex and temporal cortex from VaD and matched control patients. The dataset is available from the National Center for Biotechnology Information (NCBI) GEO database^1^ (Barrett et al., 2013). To identify overlapping DEGs, we analyzed a subset comprising 18 temporal cortex and 18 frontal cortex samples of VaD and 22 matched control samples using the Limma package in the NCBI GEO2R online tool. The threshold values for significant DEGs were adjusted *P*-value < 0.05 and | log fold change (FC)| > 1. Volcano plots were used to visualize the DEGs in frontal cortex or temporal cortex versus the matched controls. A Venn diagram was used to identify the overlapping DEGs between the two brain regions (Bardou et al., 2014).

### Gene Ontology and Pathway Enrichment Analyses

Gene ontology (GO) enrichment analysis under the three main GO categories, biological process, molecular function, and cellular component, and pathway enrichment analysis of the overlapping DEGs between the two brain regions were performed on the web-based portal Metascape^2^ (Zhou et al., 2019). The Kyoto Encyclopedia of Genes and Genomes (KEGG) (Kanehisa et al., 2017), Reactome (Jassal et al., 2020), and WikiPathways databases (Martens et al., 2021) were used for pathway annotation. The PANTHER database was applied to detect protein class over-representation of the proteins encoded by the overlapping DEGs (Mi et al., 2013). *P*-value < 0.01 was selected as the threshold for significant enrichment.

### Protein–Protein Interaction Network Construction and Module Analysis

To detect the potential relationships among the proteins encoded by the overlapping DEGs, we used the Search Tool for the Retrieval of Interacting Genes (STRING) database, version 11.0^3^ to construct a PPI network; a combined score of >0.7 was set as the cut-off criterion of statistical significance (Szklarczyk et al., 2017). Cytoscape 3.7.1^4^ was used to visualize the PPI network (Shannon et al., 2003). Ten genes with the highest degree of connectivity were identified as hub genes using the cytoHubba plugin, and key modules were identified using the molecular complex detection (MCODE) plugin.

### Hub Gene–Transcription Factor Interaction and Hub Gene–miRNA Interaction Analyses

Hub genes are considered to be key genes that play vital roles in the biological process of interest. Therefore, we performed hub gene–TF and hub gene–miRNA interaction analyses to detect TFs and miRNAs that may regulate the hub genes at transcriptional and posttranscriptional levels, respectively. The JASPAR (Khan et al., 2018) and TarBase v8 (Karagkouni et al., 2018) databases were searched to identify TFs and miRNAs based on topological parameters (i.e., degree and betweenness centrality), respectively, using the NetworkAnalyst software (Xia et al., 2015). In addition, motif analysis of DEGs was performed to discover these genes promoter sequence motifs via the MEME algorithm in the MEME Suite^5^ (Bailey et al., 2015). E-value < 0.01 was selected as the threshold for significant motifs. In order to see if any transcription factor identified above has enriched motifs on these genes, those discovered significant motifs were compared with motifs in JASPAR (NON-REDUNDANT) DNA (21 Databases) motif databases using the motif database scanning algorithm Tomtom (Gupta et al., 2007). The StarBase datasets^6^ (Li et al., 2014) presented the miRNA–target interactions by intersecting the predicting target sites of miRNAs with binding sites of Ago protein, which were derived from CLIP-seq data and users can search the interactions of miRNA-target by selecting one/multiple target-predicting programs (PITA, RNA22, miRmap, DIANA-microT, miRanda, PicTar, and TargetScan). Therefore we used it to predict microRNA binding sites of hub genes.

### Drug–Gene Interaction Analysis

The Drug--Gene Interaction Database (DGIdb)^7^ was searched to identify potential drugs that could be used in the treatment of VaD. DGIdb is an online database that provides drug-gene interaction and gene druggability information from various sources, including the literature (PubMed and clinical trial databases) and several drug databases (DrugBank, PharmGKB, and ChEMBL) (Cotto et al., 2018). The 10 identified hub genes were imported into DGIdb as potential targets to search for existing drugs that could interact with them. Drugs that showed specific types of interactions with the hub genes were selected. Cytoscape software was applied to visualize the interactions between the selected drugs and the corresponding target genes. To identify related clinical trials, the drugs that showed specific types of interactions with the hub genes were input into the ClinicalTrials.gov registry,^8^ the largest clinical trials database which contains over 329,000 trials worldwide.

## Results

### Identification of DEGs

**FIGURE 2:**
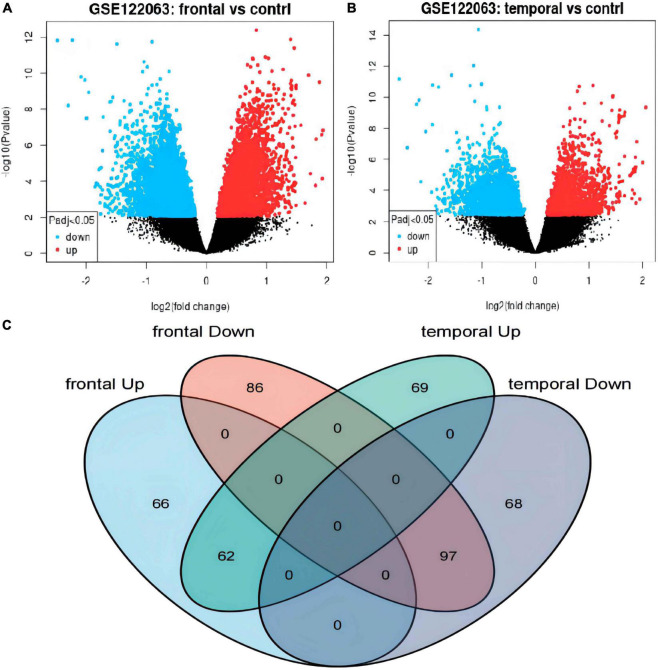
Identification of differentially expressed genes (DEGs) in frontal cortex and temporal cortex in the vascular dementia microarray dataset GSE122063. **(A,B)** Volcano plots of DEGs in frontal cortex or temporal cortex of vascular dementia versus the controls. Blue indicates downregulated genes; red indicates upregulated genes; gray indicates genes that were not significantly differentially expressed. **(C)** Venn diagram showing the numbers of overlapping DEGs between the two brain regions.

The differential expression analysis detected 128 upregulated and 183 downregulated genes in the frontal cortex (Figure 2A and Supplementary Table 1), and 131 upregulated and 165 downregulated genes in the temporal lobe cortex (Figure 2B and Supplementary Table 1) of VaD compared with the matched controls. A total of 159 overlapping DEGs, including 62 upregulated and 97 downregulated genes, were detected between the two brain regions (Figure 2C, Table 1, and Supplementary Table 1).

**TABLE 1:** Differentially expressed genes (DEGs) that overlap in the frontal cortex and temporal cortex of vascular dementia.

DEGs	Gene symbols
Upregulated DEGs (62)	*SNX31, SLAMF8, AQP1, FCGBP, SIGLEC14, VSIG4, MIA, CD163, HSPA1A, SPP1, C1QC, LILRB3, HIST1H2AC, CHORDC1, EFCAB3, HSPB1, C1QB, SLC1A7, THAP3, C6orf118, RNASE3, RNASE2, SECTM1, CCNA2, NEAT1, C5AR1, CCDC136, EBI3, STAB1, RBM14, C1QA, HSD11B2, SERPINC1, PDLIM4, PEX6, MYBPH, MS4A6A, BATF, SLC5A3, IRF7, SCIN, EIF2A, LAIR1, FCGR2C, CDH23, FLCN, DDIT4L, LILRB1, FCGR2A, FCGR3A, LAT2, ADORA3, SLC16A3, VASP, RCVRN, ITGB2, CXorf28, HMOX1, TLR2, CCR5, CSDA, TLR5*
Downregulated DEGs (97)	*SPOCK3, ZIM2, BFSP1, CHRDL2, GSTZ1, C17orf108, COLEC11, MATN3, MAEL, VAX1, C2orf82, CCNB1, CCDC113, NKAPL, PVRL3-AS1, DARC, C17orf102, GYPA, FOXC1, HTR7P1, LOC389033, QPRT, TARP, FAM178B, HRASLS5, HBA2, CYB5RL, ELOVL1, COL11A2, MAG, KLC2, LOC646999, FRMPD2, C1orf85, DRD4, BCL2L15, OR11A1, TBXA2R, RRP7B, NKX2-3, FMO1, GNG13, VGF, CLRN1-AS1, ELF2, CDKN3, FOLH1B, ZSCAN1, WDTC1, PAPL, RPL13AP17, PYCR2, DOCK3, IGF2-AS, PIGC, OPN3, C6orf221, STARD9, KCNH2, RNASE13, GPR132, KDM4D, TAC1, INSL3, C10orf27, SSR4P1, RET, ASB16, LPP-AS2, ST7-AS1, EFHB, FLT3, SUN3, SLC22A10, IL2RG, SLC35D2, TAAR5, PPEF1, OPALIN, SST, C1orf182, LIN9, OR6C74, SSX3, CRH, CARNS1, PI3, MATN2, FMO6P, KLHDC7B, GUCY2GP, KRBOX1, OR4A16, RBM3, CCT6B, LINC00458, PBOV1*

### Functional Enrichment Analysis of the Overlapping DEGs

**FIGURE 3:**
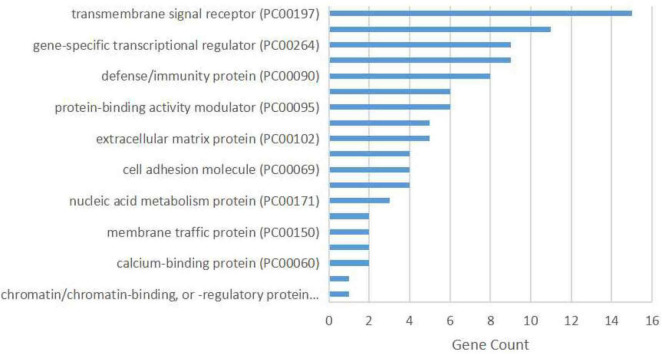
Protein class over-representation of proteins encoded by the overlapping differentially expressed genes identified using the PANTHER database.

The protein class over-representation analysis of proteins encoded by the overlapping DEGs classified them into different groups according to their functions and activities (Figure 3). The results of the GO and signal pathways enrichment analyses of the overlapping DEGs are given in Tables 2, 3, respectively. The most significantly enriched terms were inflammation and innate immunity, synapse pruning, regeneration, positive regulation of angiogenesis, response to nutrient levels, and positive regulation of the digestive system process, which may help in understanding the DEGs involved in the pathogenesis of VaD. We also performed the gene ontology enrichment and pathway enrichment analysis of up-regulated genes and down-regulated genes separately, and the results were presented in the Supplementary Materials “GO analysis” sheet and “pathway analysis” sheet of Supplementary Table 4 respectively.

**TABLE 2:** Gene ontology (GO) enrichment analysis of the overlapping differentially expressed genes in the frontal cortex and temporal cortex in vascular dementia.

Category	Term	Description	P	Symbols
Biological processes	GO:0006954	Inflammatory response	0.0000	*ADORA3, SERPINC1, C5AR1, CCR5, CRH, ACKR1, HMOX1, ITGB2, SPP1, TAC1, TBXA2R, TLR2, TLR5, CD163, STAB1, SLAMF8, NEAT1, AQP1, FMO1, PI3, RNASE2, RNASE3, VGF, LILRB1*
	GO:0002274	Myeloid leukocyte activation	0.0000	*C1QA, C5AR1, FCGR2A, HMOX1, HSPA1A, ITGB2, LAIR1, RNASE2, RNASE3, TLR2, LAT2, BATF, LILRB3, VSIG4, SIGLEC14, LILRB1, NKX2-3, CCDC136, SCIN*
	GO:0098883	Synapse pruning	0.0000	*C1QA, C1QB, C1QC, C5AR1, PI3, RNASE2, RNASE3, EBI3, VSIG4, COLEC11, ELF2, FCGR2A, FCGR3A, IRF7, ITGB2, LAT2, RBM14, LILRB1, COL11A2, BATF, LAIR1, LILRB3*
	GO:0060456	Positive regulation of digestive system process	0.0000	*AQP1, CRH, TAC1, DRD4, SLC5A3, SPP1, SLC16A3, LILRB1, CCNB1, HMOX1, HSD11B2, KCNH2, RET, SST, TBXA2R, SLAMF8, CCNA2, CCR5, FLT3, TLR2, SLC1A7, ITGB2, SCIN, ADORA3, FOXC1*
	GO:0031099	Regeneration	0.0001	*C5AR1, CCNA2, CCNB1, FLT3, HMOX1, MAG, SPP1, CDKN3, DRD4, HSPB1, RET, CHORDC1*
	GO:0002573	Myeloid leukocyte differentiation	0.0001	*C1QC, IRF7, TLR2, BATF, LILRB1, LILRB3, NKX2-3, HSPA1A, SCIN, FLT3, SLAMF8*
	GO:0002320	Lymphoid progenitor cell differentiation	0.0002	*FLT3, BATF, FLCN, LILRB1, ITGB2, TAC1, LAT2, EBI3, VSIG4, SLAMF8, NKX2-3*
	GO:0006972	Hyperosmotic response	0.0004	*AQP1, SST, YBX3, FMO1, MAG, PPEF1, TLR5, OPN3, KDM4D*
	GO:0045766	Positive regulation of angiogenesis	0.0004	*AQP1, C5AR1, HMOX1, HSPB1, ITGB2, TBXA2R, FOXC1, STAB1, CCNA2, HSPA1A, YBX3, PYCR2*
	GO:0010039	Response to iron ion	0.0005	*C1QA, CCNB1, HMOX1, AQP1, CCNA2*
	GO:0050900	Leukocyte migration	0.0006	*C5AR1, CCR5, GYPA, HMOX1, ITGB2, MAG, RET, SLC16A3, SLAMF8, NKX2-3, LAT2, FLCN, TLR2*
	GO:0001819	Positive regulation of cytokine production	0.0007	*C5AR1, HMOX1, HSPA1A, HSPB1, IRF7, TLR2, TLR5, EBI3, LILRB1, ACKR1, VSIG4, ITGB2*
	GO:0031667	Response to nutrient levels	0.0009	*SERPINC1, HMOX1, HSD11B2, SPP1, SST, TBXA2R, VGF, EIF2A, FLCN, HSPA1A, CHORDC1*
Cellular components	GO:0072562	Blood microparticle	0.0000	*SERPINC1, C1QB, C1QC, HBA2, HSPA1A, KDM4D, EIF2A*
	GO:0005788	Endoplasmic reticulum lumen	0.0003	*SERPINC1, COL11A2, FLT3, FMO1, MATN3, SPP1, VGF, EBI3, WDTC1, ASB16, FOLH1B*
Molecular functions	GO:0019864	IgG binding	0.0000	*FCGR2A, FCGR3A, FCGR2C*
	GO:0140375	Immune receptor activity	0.0001	*C5AR1, CCR5, FLT3, IL2RG, EBI3, LILRB1, FCGR3A, ITGB2, CD163, ACKR1*
	GO:0001540	Amyloid-beta binding	0.0001	*C1QA, ITGB2, TLR2, LILRB1, LILRB3, C5AR1, VSIG4, MAG, OPALIN, EBI3*
	GO:0005509	Calcium ion binding	0.0006	*MATN2, MATN3, PPEF1, RCVRN, RET, STAB1, SPOCK3, CDH23, COLEC11, SCIN, EFCAB3, EFHB*
	GO:0001530	Lipopolysaccharide binding	0.0008	*RNASE2, RNASE3, TLR2, C5AR1, PI3, COLEC11*

*The top 20 enriched GO terms are shown.*

**TABLE 3:** Molecular pathway enrichment analysis of the overlapping differentially expressed genes in frontal cortex and temporal cortex in vascular dementia.

Category	Description	p	Symbols
KEGG pathway	*Staphylococcus aureus* infection	0.0000	*C1QA, C1QB, C1QC, C5AR1, FCGR2A, FCGR3A, ITGB2, FCGR2C, SERPINC1, VSIG4, COLEC11, HSPA1A, H2AC6, TLR2, TARP*
KEGG pathway	Malaria	0.0000	*ACKR1, GYPA, HBA2, ITGB2, TLR2*
KEGG pathway	Legionellosis	0.0000	*HSPA1A, ITGB2, TLR2, TLR5, COLEC11, IRF7, IL2RG, TARP, SPP1, CCNA2, HSPB1*
KEGG pathway	Leishmania infection	0.0001	*FCGR2A, FCGR3A, ITGB2, TLR2, FCGR2C, COLEC11, RNASE3, LILRB1, LILRB3, VASP, SCIN, LAIR1*
KEGG pathway	Central carbon metabolism in cancer	0.0009	*FLT3, RET, RNASE3, SLC16A3*
Reactome	GPCR ligand binding	0.0000	*ADORA3, C5AR1, CCR5, CRH, DRD4, ACKR1, INSL3, SST, TAC1, TBXA2R, TAAR5, OPN3, GPR132, GNG13, OR11A1*
Reactome	Anti-inflammatory response favoring Leishmania parasite infection	0.0000	*CRH, FCGR2A, FCGR3A, INSL3, TAAR5, CD163, GNG13, HMOX1*
Reactome	Erythrocytes take up carbon dioxide and release oxygen	0.0000	*AQP1, HBA2, CYB5RL*
Reactome	Binding and uptake of ligands by scavenger receptors	0.0001	*HBA2, CD163, STAB1, COLEC11*
Reactome	Neutrophil degranulation	0.0014	*C5AR1, FCGR2A, HSPA1A, ITGB2, LAIR1, RNASE2, RNASE3, TLR2, SIGLEC14*
Reactome	Post-translational protein phosphorylation	0.0030	*SERPINC1, MATN3, SPP1, VGF*
Reactome	Interleukin-4 and Interleukin-13 signaling	0.0030	*HMOX1, IL2RG, ITGB2, BATF*
Reactome	Cell surface interactions at the vascular wall	0.0070	*GYPA, ITGB2, MAG, SLC16A3, SERPINC1, DOCK3, TBXA2R, GNG13, KLC2*
WikiPathways	Human complement system	0.0000	*C5AR1, FCGR3A, ITGB2, SPP1, TLR2, VSIG4, C1QC*
WikiPathways	Spinal cord injury	0.0001	*AQP1, C1QB, FCGR2A, MAG, FCGR2C, LILRB3*

*The top 15 enriched pathways are shown.*

### Protein–Protein Interaction Network Construction and Module Analysis

**FIGURE 4:**
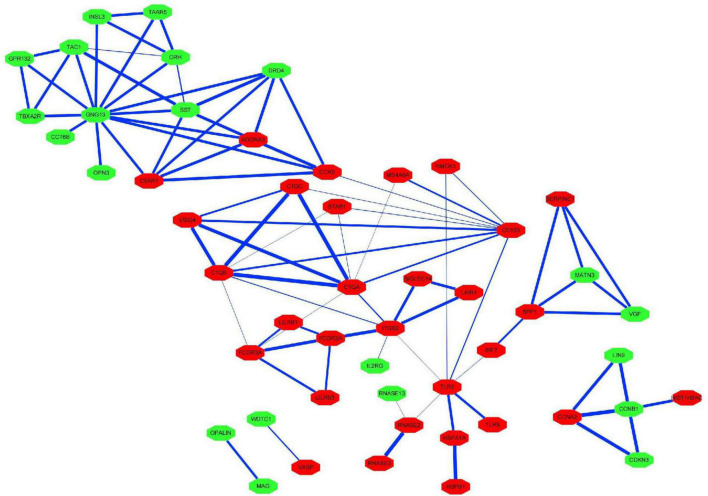
Protein–protein interaction network for the proteins encoded by the overlapping differentially expressed genes. The nodes represent the encoded proteins and the edges represent their interactions. Green indicates downregulated genes; red indicates upregulated genes. The width of the blue line indicates the combined score obtained using the STRING database.

**FIGURE 5:**
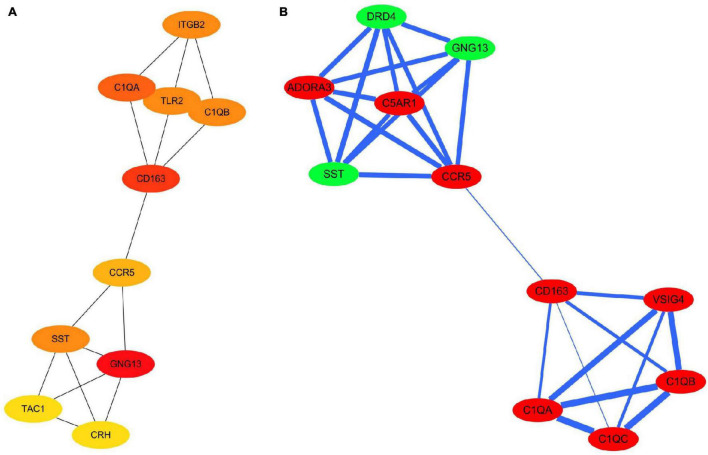
Hub genes and modules identified in the protein–protein interaction network visualized using Cytoscape software. **(A)** Top 10 hub genes with a higher degree of connectivity. **(B)** Sub-network with the highest MCODE score is shown. Green indicates downregulated genes; red indicates upregulated genes. The width of the blue line indicates the combined score obtained using the STRING database.

The PPI network of the 159 overlapping DEGs has 142 nodes and 88 edges (Figure 4 and Supplementary Table 2); disconnected nodes in the network are hidden. The average node degree of the PPI network was 1.16, and average local clustering coefficient was 0.268, and the PPI enrichment p-value was 1.82e-13. The topological analysis of the PPI network identified ten hub genes, *GNG13*, *CD163*, *C1QA*, *TLR2*, *SST*, *C1QB*, *ITGB2*, *CCR5*, *CRH*, and *TAC1* (Figure 5A and Table 4). The MCODE analysis identified three sub-networks, implying these overlapping genes may be involved in different biological processes. The sub-network with the highest MCODE score (score = 5.2) is shown in Figure 5B.

**TABLE 4:** Ten hub genes identified in the protein–protein interaction network of proteins encoded by the overlapping differentially expressed genes in vascular dementia.

Symbol	Description	Degree	Feature	References
*GNG13*	Guanine nucleotide binding protein (G protein), gamma 13	13	Playing an important role in odor-triggered social behaviors including male-male aggression and as a potential marker of the state of health of AD Patients’ cerebellum.	Liu et al., 2018; Sanfilippo et al., 2021
*CD163*	CD163 molecule	9	Promoting plaque angiogenesis, vascular permeability, inflammation, and progression of atherosclerosis.	Fabriek et al., 2005; Galea et al., 2008; Guo et al., 2018
*C1QA*	Complement component 1, q subcomponent, A chain	8	Involved in complement system regulation and plays a crucial role in neurological disorders.	Stephan et al., 2013; Lee et al., 2019
*TLR2*	Toll-like receptor 2	7	Playing a pivotal role in inflammation after ischemic brain injury and was involved in the development of diabetic microvascular complications, including endothelial dysfunction and cognitive impairment	Wang et al., 2011; Hardigan et al., 2017
*SST*	Somatostatin	7	Decreased in cerebrospinal fluid in both AD and VaD patients.	Heilig et al., 1995
*C1QB*	Complement component 1, q subcomponent, B chain	7	Involved in complement system regulation and plays a crucial role in neurological disorders	Stephan et al., 2013; Lee et al., 2019
*ITGB2*	Integrin, beta 2 (complement component 3 receptor 3 and 4 subunit)	7	Associated with atherosclerosis	Pan et al., 2020
*CCR5*	chemokine (C-C motif) receptor 5	6	Impacted learning and memory by acting on CREB signaling	Necula et al., 2021
*CRH*	Corticotropin releasing hormone	5	may be involved in the regulation of cognitive performances	Bernardi et al., 2000; Dedic et al., 2019
*TAC1*	Tachykinin, precursor 1	5	Was reported to be associated with VaD	Tian et al., 2021

### Regulatory Signatures of VaD

**FIGURE 6:**
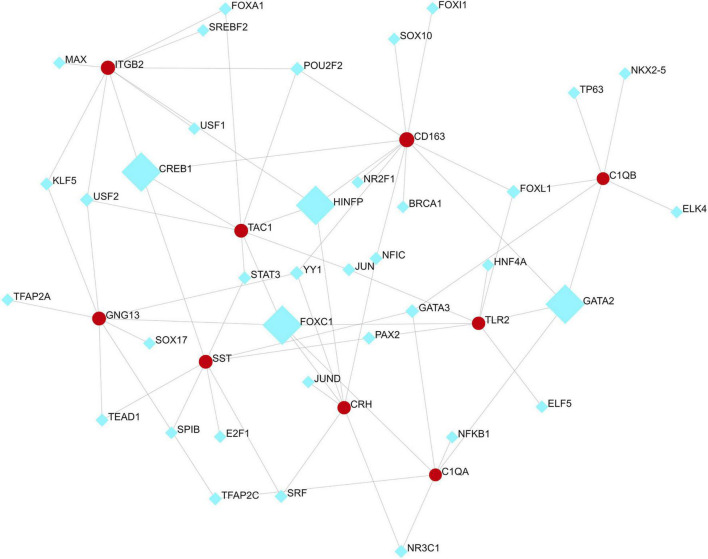
Hub gene–transcription factor interaction network. Medium confidence score was used to construct the network.

**FIGURE 7:**
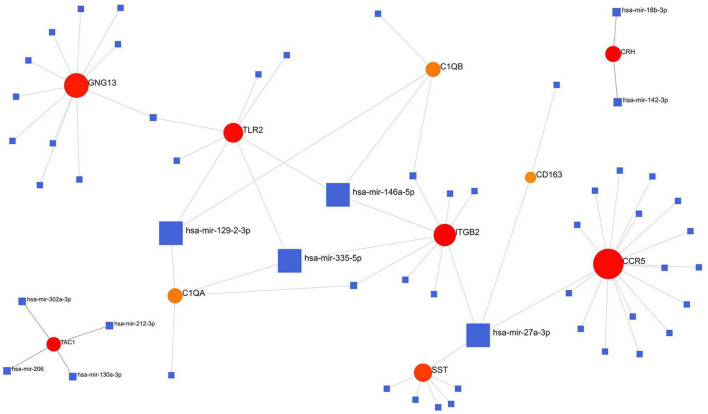
Hub gene–miRNA interaction network. Medium confidence score was used to construct the network.

**FIGURE 8:**
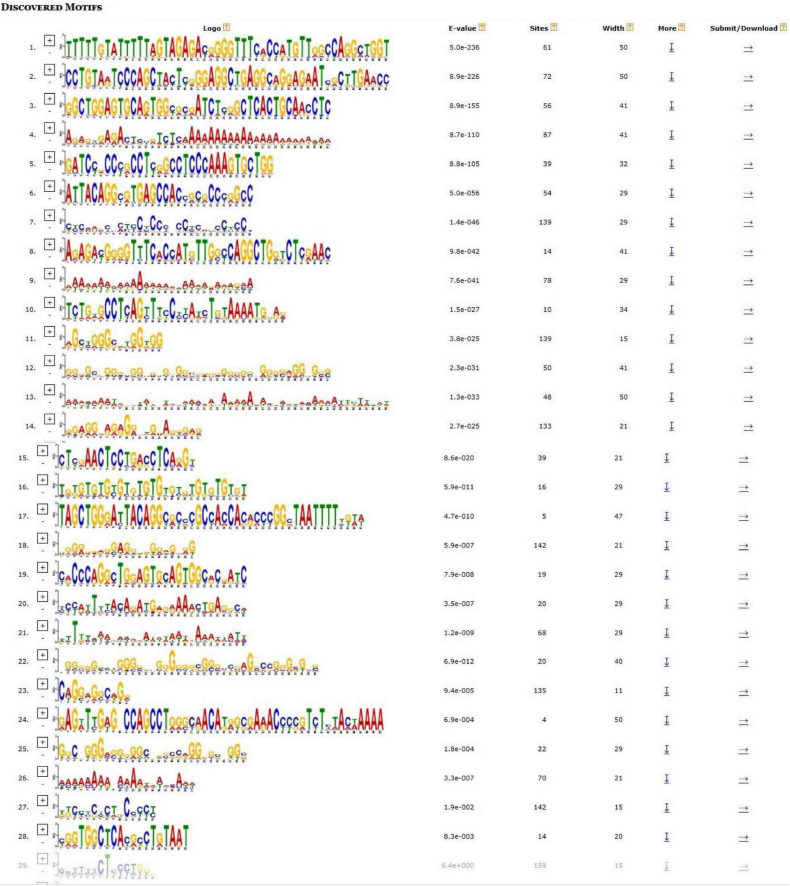
Motif analysis of differentially expressed genes (DEGs). Significant motifs were presented (E value < 0.05).

We analyzed the hub gene–TF interactions (Figure 6) and hub gene–miRNA interactions (Figure 7) and identified central regulatory TFs and miRNAs using the topological parameters. Four TFs, FOXC1, CREB1, GATA2, and HINFP, and four miRNAs, miR-27a-3p, miR-146a-5p, miR-335-5p, and miR-129-2-3p were detected from the two interaction networks, respectively (Table 5). The results of common DEGs-TF and DEGs-miRNA analysis were provided in the Supplementary Figures 1, 2, respectively, and Supplementary Table 2. We performed motif analysis of DEGs and discovered 28 significant motifs (Figure 8). And these motifs were compared with motifs in JASPAR (NON-REDUNDANT) DNA (21 Databases) motif databases and several transcription factors identified above (GATA2, TFAP2A, GATA3, MEF2A, KLF5, ELK4, SP1, JUND, EGR1, E2F6, NR2F1, NR2C2, SOX10, and E2F4) were matched to these motifs (Supplementary Table 5). The results of the microRNA binding sites of these hub genes were provided in the Supplementary Table 6.”

**TABLE 5:** Transcription factors (TFs) and miRNAs identified as transcriptional and/or posttranscriptional regulators of the hub genes in the protein–protein interaction network of VaD.

Symbol	Description	Degree	Betweenness	Feature	References
FOXC1	Forkhead box C1	5	130.93	Involved in processes of vascular development, such as in arterial specification and angiogenesis regulation.	Tan and Markus, 2015
CREB1	Cyclic adenosine monophosphate (cAMP) responsive element-binding protein1	4	113.16	Afflicted with VaD	Han et al., 2018
GATA2	GATA binding protein 2	4	112.13	Afflicted with AD, the role in VaD was not known.	Rahman M. R. et al., 2020
HINFP	Histone H4 transcription factor	4	75.42	May be involed in type 2 diabetes and AD.	Rahman M. H. et al., 2020
**miRNAs**					
mir-27a-3p	MicroRNA 27	4	898	Afflicted with atherosclerosis	Choe et al., 2020
mir-146a-5p	MicroRNA 146	3	318.8	Afflicted with atherosclerosis and endothelial inflammation	Lo et al., 2017; La Sala et al., 2019
mir-335-5p	MicroRNA 335	3	318.8	Playing a critical role in spatial learning and synaptic plasticity	Capitano et al., 2017
mir-129-2-3p	MicroRNA 129	3	46.93	Significantly lower in ischemic stroke patients and negatively associated with the risk of ischemic stroke	Huang et al., 2019

*Hub gene–TF and hub gene–miRNAs interactions were analyzed.*

### Drug–Gene Interaction Analysis

**FIGURE 9:**
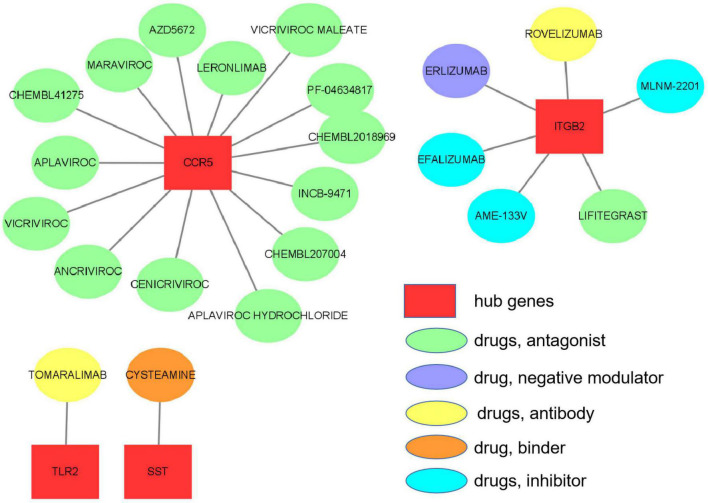
Hub gene–drug interactions that were found in the Drug–Gene Interaction Database (DGIdb) for four hub genes, *TLR2*, *SST*, *ITGB2*, and *CCR5*, are visualized using Cytoscape software.

The 10 hub genes were considered as potential druggable targets for VaD treatment. The drug-gene interaction analysis found 69 candidate target drugs/compounds for VaD treatment. Among them, 22 targeted *TAC1*, 19 targeted *ITGB2*, 15 targeted *CCR5*, 7 targeted *SST*, 4 targeted *TLR2*, and 2 targeted *CRH*. For 47 of the candidate drugs, no specific types of interactions with the hub genes have been reported, and therefore these drugs need further investigation. For the other 22 candidate drugs, specific types of interactions with hub genes have been reported and were visualized using Cytoscape software (Figure 9 and Supplementary Table 3). No candidate drugs were identified for *GNG13*, *CD163*, *C1QA*, or *TLR2*. Until now, none of the 22 drugs have been used directly to treat VaD, as was shown by searching the ClinicalTrials.gov registry.

## Discussion

Vascular dementia is a progressive disease that affects cognitive abilities, especially executive functioning. The heterogeneity of causes of VaD makes it challenging to elucidate the neuropathological substrates and mechanisms of VaD. In this study, we used multi-stage bioinformatics analyses to identify biomolecular signatures that underlie the pathophysiological mechanisms of VaD by analyzing the gene expression patterns in the temporal cortex and frontal cortex samples from patients with VaD.

A total of 159 overlapping DEGs between these brain regions were identified. We performed GO and pathway enrichment analysis to obtain further insights into the functions and signaling pathways of the overlapping DEGs. We found that these DEGs were enriched mainly in inflammation and innate immunity, synapse pruning, regeneration, positive regulation of angiogenesis, response to nutrient levels, and positive regulation of the digestive system process. Accumulating evidence indicates that inflammation and innate immunity play crucial roles in the progression of VaD. Synaptic pruning is essential for the development and maintenance of healthy brain circuitry by removing less active or “weak” synapses to allow the strengthening and maturation of more active connections in a neural activity-dependent process. Disruption of synaptic pruning has been associated with several neural disorders, such as schizophrenia and AD (Presumey et al., 2017). The synaptic pruning pathway was shown to be activated early in the brain of patients with AD where it mediates synapse loss (Hong et al., 2016), yet its role in VaD is largely unknown and needs future research. Increasing evidence has suggested that aberrant angiogenesis may play a role in the development of VaD, but its precise role remains controversial. A significant association between angiogenesis activity and cerebrovascular disease, which is a significant contributor to VaD, has been reported (Callahan et al., 2020), whereas other studies have indicated that promoting angiogenesis, especially in functional blood vessels, can reduce the extent of ischemia and improve cognition in VaD (Yang et al., 2017; Zhao et al., 2019). Clearly, more studies are needed to better understand the potential of angiogenesis as an intervention target.

We performed PPI analysis to discover the potential relationships among the proteins encoded by the overlapping DEGs and identified 10 hub genes (*GNG13*, *CD163*, *C1QA*, *TLR2*, *SST*, *C1QB*, *ITGB2*, *CCR5*, *CRH*, and *TAC*). Heterotrimeric G proteins, which consist of alpha, beta, and gamma subunits, function as signal transducers for the seven-transmembrane, G protein-coupled receptors. *GNG13*, which encodes a gamma subunit, is expressed in taste, retinal, and neuronal tissues, and the GNG13 protein is involved in the gustatory signal transduction pathway by interacting with proteins containing PDZ domains (Li et al., 2006; Liu et al., 2012). Liu et al. (2018) suggested that the GNG13 subunit was a critical signaling component in both the main olfactory epithelium and apical vomeronasal epithelium, by playing an important role in odor-triggered social behaviors including male–male aggression. A recent study found the expression level of *GNG13* was significantly reduced in patients with AD compared with its expression in healthy control subjects and *GNG13* may act as a potential biomarker in Purkinje cells, indicating the state of health of the cerebellum (Sanfilippo et al., 2021). However, insufficient information is available on its role in VaD. Future experiments are needed for verification.

The protein encoded by *CD163* is a cell-surface glycoprotein in the scavenger receptor cysteine-rich superfamily, and it is expressed exclusively in monocytes and macrophages (Ritter et al., 1999). A recent study found that *CD163*+ macrophages promoted plaque angiogenesis, vascular permeability, inflammation, and progression of atherosclerosis, and deletion of *CD163* in mice reduced intraplaque neovascularization and plaque progression (Guo et al., 2018). Besides, *CD163*+ macrophages and microglia were in the central and peripheral nervous system and may play a role in the inflammatory process (Fabriek et al., 2005; Galea et al., 2008). Atherosclerosis and inflammatory responses are known to be related to VaD (Kalaria, 2018), which is consistent with our results. The proteins encoded by *C1QA* and *C1QB* belong to the C1q family, whose members are the first components of the complement pathway and are involved in complement system regulation and play crucial roles in neurological disorders (Lee et al., 2019). A previous study indicated that aged *C1qa*-knockout mice showed reduced levels of cognitive and memory decline (Stephan et al., 2013), which is in accordance with the results of our study.

The protein encoded by *TLR2* is a member of the toll-like receptor (TLR) family, whose members are significant pattern recognition receptors of the innate immune system, initiating inflammatory cascades by recognizing pathogen- and damaged-associated molecular patterns. TLR2 was found to play a pivotal role in inflammation after ischemic brain injury (Wang et al., 2011) and was involved in the development of diabetic microvascular complications, including endothelial dysfunction and cognitive impairment (Hardigan et al., 2017). In this study, we found that *TLR2* was upregulated in the VaD samples compared with the controls samples, which supports the previous results.

Somatostatin, which is encoded by *SST*, is a widely distributed peptide in the central nervous system where it affects the rates of neurotransmission, and it was reported to be decreased in cerebrospinal fluid in patients with AD and VaD (Heilig et al., 1995). *CCR5* encodes a seven-membrane, G protein-coupled receptor that may impact learning and memory by acting on CREB signaling, a pathway that is critical for learning and memory (Necula et al., 2021). *CRH* encodes a member of the corticotropin-releasing factor family that functions as a significant regulator of homeostasis, mediating the autonomic, behavioral, and neuroendocrine responses to stress (Dedic et al., 2019). Previous studies indicated that CRH might be involved in the regulation of cognitive performances (Bernardi et al., 2000).

Hub genes are considered to be key genes that play vital roles in biological processes and can affect the regulation of other genes in related pathways; thus, hub genes are often important targets and research hotspots. We studied hub gene–TF and hub gene–miRNA interactions to identify potential transcriptional and posttranscriptional regulators of the 10 identified hub genes. We identified four TFs (FOXC1, CREB1, GATA2, and HINFP) and four miRNAs (miR-27a-3p, miR-146a-5p, miR-335-5p, and miR-129-2-3p) as regulators of the hub genes in VaD. FOXC1 is involved in vascular development processes, including arterial specification and angiogenesis regulation, and may play a role in small vessel disease, which is the primary pathology underlying vascular cognitive impairment (Tan and Markus, 2015). Cyclic adenosine monophosphate responsive element-binding protein 1 (CREB1) is a leucine-zipper transcription factor that plays an essential role in long-term memory formation (Sadamoto et al., 2010). Silencing of *CREB1* exasperated cognitive dysfunction in vascular dementia (VD) mouse model by inhibiting activation of the PKA-CREB signaling pathway (Han et al., 2018). *GATA2*, which encodes GATA-binding protein 2, was found to be differentially expressed in AD (Rahman M. R. et al., 2020), but its role in VaD has not been reported so far.

The reduced level of miR-27a-3p found in the cerebrospinal fluid of patients with AD indicated it as a candidate biomarker for AD (Sala et al., 2013). A recent study reported that reduction of miR-27a-3p in vascular smooth muscle cells may result in the development of vascular calcification that develops in association with atherosclerosis, one of the critical risk factors of VaD (Choe et al., 2020). miR-146a-5p was also reported to be associated with dysfunction of the vascular endothelium and may be involved in regulating high glucose-induced endothelial inflammation and atherosclerosis (Lo et al., 2017; La Sala et al., 2019). Capitano et al. (2017) found that overexpression of miR-335-5p impaired spatial memory and long-term potentiation maintenance in mice, indicating that miR-335-5p may play a critical role in spatial learning and synaptic plasticity. The blood level of miR-129-2-3p was significantly lower in ischemic stroke patients and negatively associated with the risk of ischemic stroke (Huang et al., 2019; Lo et al., 2017). These miRNAs can be considered as candidate biomarkers for VaD.

To the best of our knowledge, there are no definitive drugs available for VaD treatment. We performed a drug-hub gene interaction analysis to detect potential target drugs/compounds for VaD treatment and identified a total of 22 drugs that were predicted to have specific types of interactions with the hub genes. We checked the 22 candidate drugs in the ClinicalTrials.gov registry (see footnote 8). Although none of the 22 drugs have been used directly to treat VaD, four of them (maraviroc, cenicriviroc, PF-04634817, and efalizumab) could be potential drugs for VaD treatment. Maraviroc, cenicriviroc, and PF-04634817 are all CCR5 antagonists and efalizumab is an IGTB2 antagonist. Maraviroc and cenicriviroc were reported to improve cognition in HIV-infected individuals with cognitive impairment through their antiretroviral and anti-inflammatory effects (Mora-Peris et al., 2018; Alagaratnam et al., 2019). Moreover, maraviroc was found to reduce cardiovascular risk by modulation of atherosclerotic progression *in vivo* and *in vitro* (Afonso et al., 2017; Francisci et al., 2019). PF-04634817 and efalizumab are being developed for the treatment of diabetic nephropathy (Gale et al., 2018) and type 1 diabetes mellitus (Posselt et al., 2010), respectively. Diabetes mellitus and its complications are known risk factors for VaD. These four drugs need to be evaluated as potential drugs in VaD treatment in future studies.

Our study has some limitations. First, our results are based on publicly available microarray data, and no clinical or experimental confirmation of the roles of the proteins encoded by the identified genes of interest was attempted in the present study. Second, the expression profiles from only one GEO dataset were analyzed because of the limited number of available microarray datasets of VaD. In future studies, if available, multiple datasets should be analyzed simultaneously to increase the reliability of the results. Notwithstanding these limitations, this study offers some insights into the underlying mechanisms involved in the progression of VaD.

## Conclusion

We identified overlapping DEGs between the frontal cortex and temporal cortex of VaD. These overlapping DEGs were enriched mainly in GO terms and pathways associated with inflammation and innate immunity, synapse pruning, regeneration, positive regulation of angiogenesis, and response to nutrient levels, all of which play crucial roles in the progression of VaD. We also identified hub genes, TFs, and miRNAs that were predicted to regulate the expression of the hub genes, as well as candidate drugs that target the hub genes. Our results may contribute to understanding the underlying mechanisms in VaD and provide potential targets and drugs for therapeutic intervention.

## Data Availability Statement

The datasets presented in this study can be found in online repositories. The names of the repository/repositories and accession number(s) can be found in the article/Supplementary Material.

## Author Contributions

JS, WW, and LZ conceived and designed the study, analyzed the data, wrote the manuscript, and contributed to the designs of the methods used in this study. All authors have read and agreed to the publication of this version of the manuscript.

## Conflict of Interest

The authors declare that the research was conducted in the absence of any commercial or financial relationships that could be construed as a potential conflict of interest. The reviewer YZ has declared a shared parent affiliation with the authors at the time of review.

## Publisher’s Note

All claims expressed in this article are solely those of the authors and do not necessarily represent those of their affiliated organizations, or those of the publisher, the editors and the reviewers. Any product that may be evaluated in this article, or claim that may be made by its manufacturer, is not guaranteed or endorsed by the publisher.

## Footnotes

1
https://www.ncbi.nlm.nih.gov/geo/

2
http://metascape.org/

3
www.string-db.org

4
www.cytoscape.org

5
http://meme-suite.org

6
http://starbase.sysu.edu.cn/

7
www.dgidb.org

8
https://clinicaltrials.gov/
