# Supplementary material for: Identification of Molecular Signatures and Candidate Drugs in Vascular Dementia by Bioinformatics Analyses
Source: Front Mol Neurosci. 2022 Feb 11;15:751044. doi: 10.3389/fnmol.2022.751044 (PMC8873373; doi:10.3389/fnmol.2022.751044)
Supplement: Supplementary file 3 [file Table_3.DOCX]

Supplementary Material

# Supplementary Data

Fig. S1. Common differentially expressed genes–transcription factor interaction network. Medium confidence score was used to construct the network.

Fig. S2. Common differentially expressed genes–miRNA interaction network. Medium confidence score was used to construct the network.

**Supplementary Table S7**

Table S1. Details of the drug–hub gene interactions detected by mapping the ten hub genes to the Drug–Gene Interaction Database (DGIdb).

| Gene symbol | Gene name | Target agent | Drug-gene Interaction type（Interaction Type & Directionality） | Query Score | Interaction Score |
| --- | --- | --- | --- | --- | --- |
| TLR2 | toll-like receptor 2 | TOMARALIMAB | antibody (inhibitory) | 8.77 | 31.9 |
|  |  | CHEMBL1836411 | N/A | 8.77 | 31.9 |
|  |  | RESVERATROL HEXANOIC ACID | N/A | 8.77 | 31.9 |
|  |  | DIAPEP-277 | N/A | 2.19 | 7.97 |
| SST | somatostatin | CYSTEAMINE | binder | 8.77 | 18.23 |
|  |  | VALINOMYCIN | N/A | 4.38 | 9.11 |
|  |  | GANCICLOVIR | N/A | 2.19 | 4.56 |
|  |  | CAPTOPRIL | N/A | 0.67 | 1.4 |
|  |  | AMPHETAMINE | N/A | 0.58 | 1.22 |
|  |  | STREPTOZOCIN | N/A | 0.19 | 0.4 |
|  |  | LITHIUM | N/A | 0.19 | 0.4 |
| ITGB2 | integrin, beta 2 (complement component 3 receptor 3 and 4 subunit) | MLNM-2201 | inhibitor (inhibitory) | 4.38 | 3.36 |
|  |  | ERLIZUMAB | negative modulator (inhibitory) | 4.38 | 3.36 |
|  |  | BUTEIN | N/A | 4.38 | 3.36 |
|  |  | ROVELIZUMAB | antibody (inhibitory), antagonist (inhibitory) | 2.92 | 2.24 |
|  |  | EFALIZUMAB | inhibitor (inhibitory) | 2.19 | 1.68 |
|  |  | AME-133V | inhibitor (inhibitory) | 2.19 | 1.68 |
|  |  | LIFITEGRAST | antagonist (inhibitory) | 1.46 | 1.12 |
|  |  | MYCOPHENOLATE MOFETIL | N/A | 1.46 | 1.12 |
|  |  | ANTIBIOTIC | N/A | 0.88 | 0.67 |
|  |  | SODIUM CHLORIDE | N/A | 0.63 | 0.48 |
|  |  | METHYLPREDNISOLONE | N/A | 0.55 | 0.42 |
|  |  | PREDNISONE | N/A | 0.27 | 0.21 |
|  |  | COLCHICINE | N/A | 0.27 | 0.21 |
|  |  | PENTOXIFYLLINE | N/A | 0.26 | 0.2 |
|  |  | THALIDOMIDE | N/A | 0.25 | 0.19 |
|  |  | INDOMETHACIN | N/A | 0.23 | 0.18 |
|  |  | CYCLOSPORINE | N/A | 0.21 | 0.16 |
|  |  | CYCLOPHOSPHAMIDE | N/A | 0.11 | 0.08 |
|  |  | ALCOHOL | N/A | 0.1 | 0.08 |
| CCR5 | chemokine (C-C motif) receptor 5 | MARAVIROC | antagonist (inhibitory) | 28.5 | 27.64 |
|  |  | LERONLIMAB | antagonist (inhibitory), antibody (inhibitory) | 21.92 | 21.26 |
|  |  | VICRIVIROC | antagonist (inhibitory) | 21.92 | 21.26 |
|  |  | CHEMBL207004 | antagonist (inhibitory) | 17.54 | 17.01 |
|  |  | INCB-9471 | antagonist (inhibitory) | 8.77 | 8.51 |
|  |  | CENICRIVIROC | antagonist (inhibitory) | 8.77 | 8.51 |
|  |  | AZD5672 | antagonist (inhibitory) | 8.77 | 8.51 |
|  |  | ANCRIVIROC | antagonist (inhibitory) | 4.38 | 4.25 |
|  |  | VICRIVIROC MALEATE | antagonist (inhibitory) | 4.38 | 4.25 |
|  |  | APLAVIROC | antagonist (inhibitory) | 4.38 | 4.25 |
|  |  | PF-232798 | N/A | 4.38 | 4.25 |
|  |  | APLAVIROC HYDROCHLORIDE | antagonist (inhibitory) | 2.19 | 2.13 |
|  |  | CHEMBL41275 | antagonist (inhibitory) | 2.19 | 2.13 |
|  |  | PF-04634817 | antagonist (inhibitory) | 2.19 | 2.13 |
|  |  | CHEMBL2018969 | antagonist (inhibitory) | 2.19 | 2.13 |
| CRH | corticotropin releasing hormone | NALOXONE | N/A | 0.8 | 5.8 |
|  |  | ROSIGLITAZONE | N/A | 0.34 | 2.45 |
| TAC1 | tachykinin, precursor 1 | NIZATIDINE | N/A | 4.38 | 2.9 |
|  |  | GINGER | N/A | 4.38 | 2.9 |
|  |  | MEXILETINE | N/A | 2.19 | 1.45 |
|  |  | FAMOTIDINE | N/A | 2.19 | 1.45 |
|  |  | ETHER | N/A | 1.46 | 0.97 |
|  |  | METHOXSALEN | N/A | 1.1 | 0.72 |
|  |  | PUROMYCIN | N/A | 1.1 | 0.72 |
|  |  | NALTREXONE | N/A | 1.1 | 0.72 |
|  |  | ETIDRONIC ACID | N/A | 0.97 | 0.64 |
|  |  | BACLOFEN | N/A | 0.88 | 0.58 |
|  |  | LORATADINE | N/A | 0.82 | 0.54 |
|  |  | FUROSEMIDE | N/A | 0.67 | 0.45 |
|  |  | TERFENADINE | N/A | 0.58 | 0.39 |
|  |  | RESERPINE | N/A | 0.53 | 0.35 |
|  |  | SULINDAC | N/A | 0.49 | 0.32 |
|  |  | CHLORHEXIDINE | N/A | 0.46 | 0.31 |
|  |  | OMEPRAZOLE | N/A | 0.39 | 0.26 |
|  |  | PROTOPORPHYRIN | N/A | 0.35 | 0.23 |
|  |  | DIGOXIN | N/A | 0.26 | 0.17 |
|  |  | WORTMANNIN | N/A | 0.24 | 0.16 |
|  |  | GOSSYPOL | N/A | 0.18 | 0.12 |
|  |  | HALOPERIDOL | N/A | 0.12 | 0.08 |
| Drug–gene Interaction types are as follows: binder, the drug physically binds to the target; inhibitor, the drug binds to a target and decreases its expression or activity. (Most interactions of this type are enzyme inhibitors that bind to an enzyme to reduce its activity [Directionality: inhibitor].); negative modulator, the drug negatively regulates the amount or activity of the target. (Unlike an inhibitory allosteric modulator, a negative modulator may not directly bind to the target [Directionality: inhibitory].); antagonist, the drug blocks or dampens agonist-mediated responses rather than producing a biological response itself upon binding to a target receptor (Directionality: inhibitory); N/A, DGIdb assigns this label to any drug–gene interaction for which the interaction type is not specified by the reporting source. | | | | | |
