# Supplementary figures and images for: Identification of Molecular Signatures and Candidate Drugs in Vascular Dementia by Bioinformatics Analyses
Source: Front Mol Neurosci. 2022 Feb 11;15:751044. doi: 10.3389/fnmol.2022.751044 (PMC8873373; doi:10.3389/fnmol.2022.751044)

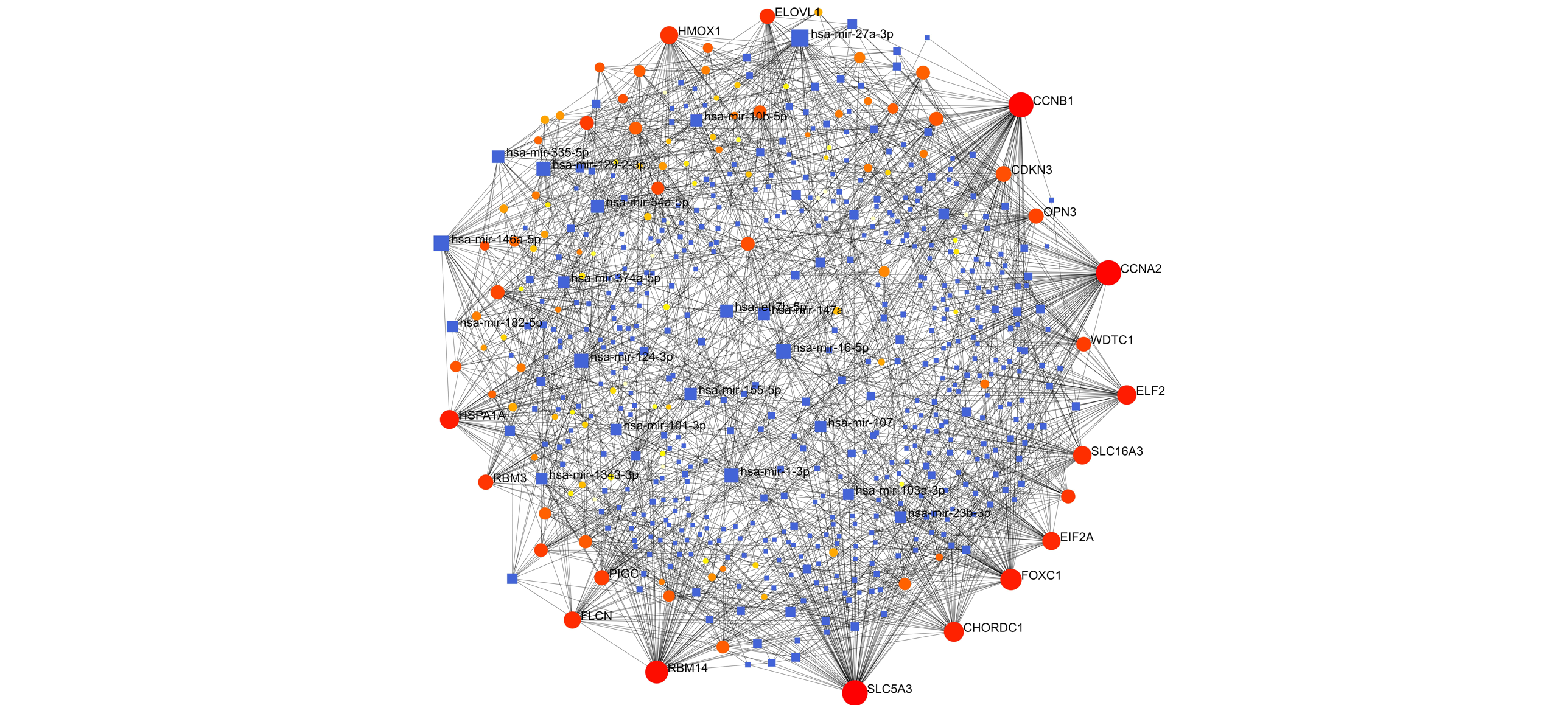

Supplement: Supplementary file 7 [file Data_Sheet_1.ZIP › FigureS2 300DPI.jpg]
